# Calendar

**DOI:** 10.1155/S1463924685000116

**Published:** 1985


### Calendar

### APRIL 1985

### Fourier Transform Infra-red Spectroscopy

Joint meeting of the Northern Ireland Region of the Analytical Division of the Royal Society of Chemistry and the Ireland Region of the RSC. To be held on 17 April at Queen's University, Belfast, UK.

*Further information from the Analytical Division, Royal Society of Chemistry, Burlington House, London W1V 0BN. Tel: 01 734 9971.*

### Automated Methods in Pharmaceutical Analysis

Meeting of the North East Region of the Analytical Division of the Royal Society of Chemistry. Six speakers including K. J. Leiper on 'Robotics'. To be held on 17 April at Newcastle Polytechnic.

*Further information from the Analytical Division, RSC (above).*

### Robotics in the Laboratory

A meeting of the North West Region of the Analytical Division, Royal Society of Chemistry, together with the Society of Chemical Industry, Liverpool Section, and the Stanlow Branch of the Institute of Petroleum. To be held at the Thornton Research Centre, Shell Research Ltd, near Chester, UK on Friday 19 April 1985.

*Further information from the Analytical Division, RSC (above).*

### Quality-control in the Chemical Laboratory—The National Testing Laboratory Accreditation Scheme

A joint meeting of the Western Region of the Analytical Division and the Mid-Southern Counties Section of the Royal Society of Chemistry to be held at 3 p.m. on Friday 26 April 1985, at BDH Ltd, Broom Road, Poole, UK. The speaker will be E. Broderick, Chemist responsible for Accreditation of Chemistry Laboratories at the National Physical Laboratory.

*Further information from the Analytical Division, RSC (above).*

### Microprocessor Familiarization

Course to be held from 22–23 April in Bromley, UK.

*Further information from Sira Ltd, South Hill, Chislehurst, Kent BR7 5EH, UK.*

### Congress of Laboratory Medicine

To be held from 27 April to 2 May in Hamburg, FR Germany.

*Further information from Hamburg Messe und Congress GmbH, Kongress für Laboratoriumsmedizin, Postfach 30 24 80. D 200 Hamburg 36, FR Germany.*

### MAY 1985

### 6th ECCLS Seminar

To be held from 8 to 10 May in Cologne, FR Germany.

*Further information from ECCLS Central Office, Wellcome Research Laboratories, Beckenham, Kent BR3 3BS, UK.*

### Moisture Measurement in Solids and Gases

Course to be held from 14 to 15 May in Chislehurst, UK.

*Further information from Sira (above).*

### JUNE 1985

### Hewlett-Packard 1985 Analytical Symposium

To be held from 10 to 14 June 1985 at the Moat House, Stratford-upon-Avon, UK.

*Further information from Mrs Tina Mears, Hewlett-Packard Ltd, Miller House, The Ring, Bracknell, Berkshire, UK.*

### Product Design Assurance in Engineering

To be held from 11 to 13 June 1985 at the Wembley Conference Centre, London.

*Further information from Mrs H. M. W. Gibbons, Society of Environmental Engineers, Owles Hall, Buntingford, Hertfordshire, UK.*

### Computing in Clinical Laboratories

To be held from 12 to 14 June in Stuttgart, FR Germany.

*Further information from The Chairman of the Fifth Conference CCL '85, PD Dr Chr Trendelenburg, Katharinenhospital, Klinisch-Chemisches Institut, Kriegsbergstraße 60, D-7000 Stuttgart 1, FR Germany.*

### Developments in Analytical Methods in the Pharmaceutical and Biomedical Sciences

Organized by the Italian Group for Mass Spectrometry in Biochemistry and Medicine and by the Department of Chemical Sciences of the University of Camerino. To be held in Camerino, Italy from 17 to 20 June.

*Further information from Dr Alberto Frigerio, Italian Group for Mass Spectrometry in Biochemistry and Medicine, Via Eustachi, 36 20129, Milan, Italy.*

